# Phloretin attenuates STAT-3 activity and overcomes sorafenib resistance targeting SHP-1–mediated inhibition of STAT3 and Akt/VEGFR2 pathway in hepatocellular carcinoma

**DOI:** 10.1186/s12964-019-0430-7

**Published:** 2019-10-16

**Authors:** Sarita Saraswati, Abdulqader Alhaider, Abdelgalil Mohamed Abdelgadir, Pooja Tanwer, Hesham M. Korashy

**Affiliations:** 1 Department of Pharmacology and Physiology, College of Medicine, |King Saud University, Riyadh, Kingdom of Saudi Arabia; 2 Department of Basic Medical Sciences, College of Medicine, King Saud bin Abdulaziz University for Health Sciences, Riyadh, Kingdom of Saudi Arabia; 3 Department of Biochemical Engineering and Biotechnology, Indian Institute of Technology, Hauz Khas-New Delhi, India; 4 Department of Pharmaceutical Sciences, College of Pharmacy, Qatar University, Doha, 2713 Qatar

**Keywords:** Phloretin, Sorafenib, STAT3, SHP-1, hepatocellular carcinoma

## Abstract

**Background:**

Hepatocellular carcinoma (HCC) is the most common primary liver malignancy. Phloretin (PH) possesses anticancer, antitumor, and hepatoprotective effects, however, the effects and potential mechanisms of phloretin remain elusive.

**Methods:**

Five HCC cells were tested in vitro for sensitivity to PH, Sorafenib (Sor) or both and the apoptosis, signal transduction and phosphatase activity were analyzed. To validate the role of SHP-1, we used PTP inhibitor III and SHP-1 siRNA. Further, we used purified SHP-1 proteins or HCC cells expressing deletion N-SH2 domain or D61A point mutants to study the PH efficacy on SHP-1. The `in vivo studies were conducted using HepG2 and SK-Hep1 and Sor resistant HepG2^SR^ and Huh7^SR^ xenografts. Molecular docking was done with Swiss dock and Auto Dock Vina.

**Results:**

PH inhibited cell growth and induced apoptosis in all HCC cells by upregulating SHP-1 expression and downregulating STAT3 expression and further inhibited pAKT/pERK signaling. PH activated SHP-1 by disruption of autoinhibition of SHP-1, leading to reduced p-STAT3^Tyr705^ level. PH induced apoptosis in two Sor-resistant cell lines and overcome STAT3, AKT, MAPK and VEGFR2 dependent Sor resistance in HCCs. PH potently inhibited tumor growth in both Sor-sensitive and Sor-resistant xenografts in vivo by impairing angiogenesis, cell proliferation and inducing apoptosis via targeting the SHP-1/STAT3 signaling pathway.

**Conclusion:**

Our data suggest that PH inhibits STAT3 activity in Sor-sensitive and -resistant HCCs via SHP-1–mediated inhibition of STAT3 and AKT/mTOR/JAK2/VEGFR2 pathway. Our results clearly indicate that PH may be a potent reagent for hepatocellular carcinoma and a noveltargeted therapy for further clinical investigations.

**Graphical abstract:**

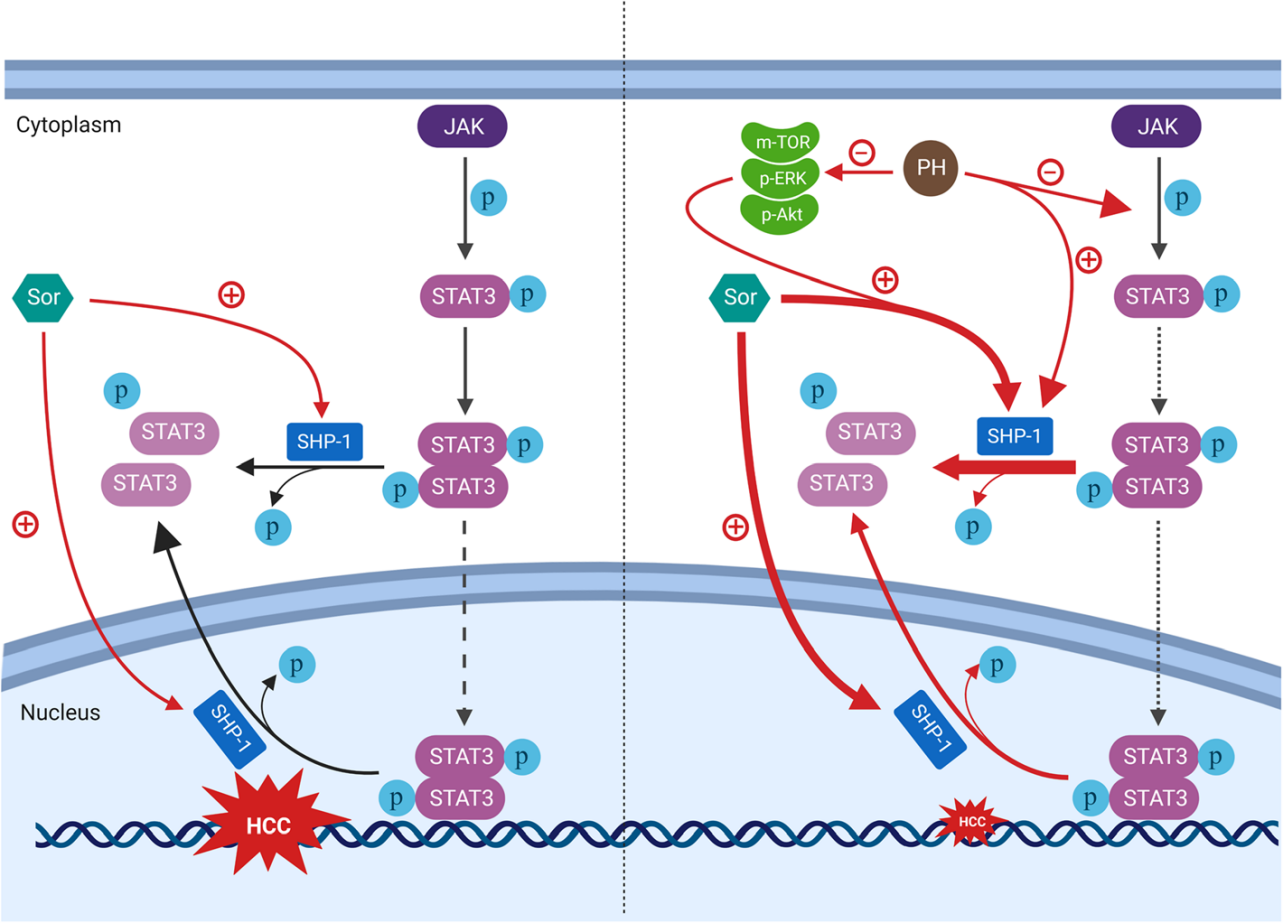

**Electronic supplementary material:**

The online version of this article (10.1186/s12964-019-0430-7) contains supplementary material, which is available to authorized users.

## Background

**Fig. 1:**
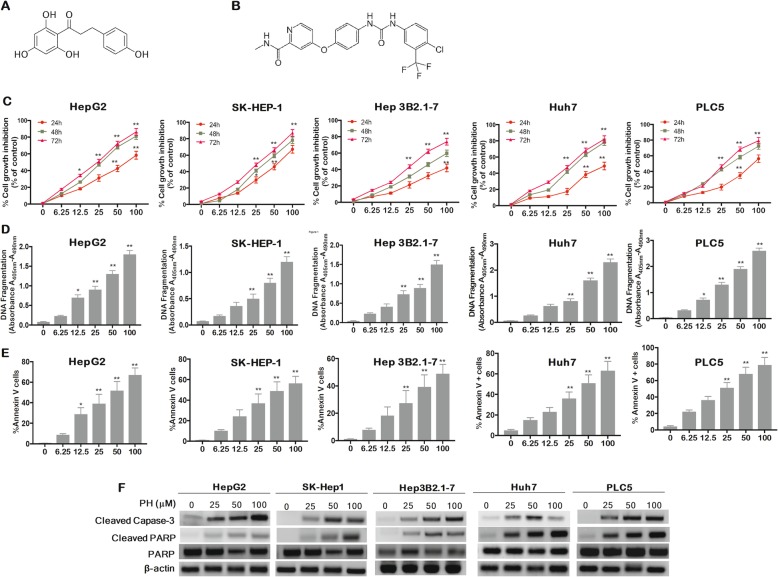
PH inhibits cell proliferation and induces apoptosis in HCCs. **a** Sor and (**b**) PH structure. **c** Cell viability by MTT assay. **d** DNA fragmentation assay. **e** Apoptosis was analyzed by annexin V, FACS analysis. Annexin V (+) cells were quantified. **f** The protein levels of caspase-3 and PARP were determined by Western blot after exposing HCCs to PH for 48 h. Experiments were conducted in triplicate and mean values ± SD (bars) are shown. *p < 0.05, and **p < 0.01 compared to control

Hepatocellular carcinoma (HCC) is the fifth leading cause of cancer death worldwide accounting for 9.1% deaths [1]. Sorafenib (Sor, Fig. 1a), a tyrosine kinase inhibitor, is the first and only targeted drug approved for use in HCC [2]. Although, it prolongs the median survival time with limited side effects in HCC patients, many patients become resistant to Sor, which is the bottleneck in extending the overall survival time for HCC patients [3]. Signal Transducer and Activator of Transcription 3 (STAT3) represents a key transducer in signaling pathways involved in the injury-inflammation-regeneration response associated with chronic liver diseases and human HCC development [4, 5]. STAT3 is considered as a major target by Sor [4, 5], in which increased STAT3 activity is related to the poor diagnosis, severe drug resistance and declining survival period in patients [6].

The Src homology region 2 domain-containing phosphatase-1 (SHP-1) plays a crucial role in glucose homeostasis and lipid metabolism in the liver [7, 8]. SHP-1 is one of the protein tyrosine phosphatase (PTP) members that could suppress STAT3 pathway [9] and dephosphorylate JAK kinases [10] and STAT3 directly [11]. SHP-1 has been shown to function as a tumor suppressor to inhibit the tumor growth [12, 13]. Certain target drugs such as dovitinib and SC-2001 are known to induce apoptosis, autophagy, and HCC cell growth inhibition through enhancing the activity of SHP-1 tyrosine phosphatase in HCC [14, 15]. Various STAT3 inhibitors have been designated to directly target STAT3 mainly by inhibiting its dimerization, DNA binding, or nuclear translocation [16, 17]. However, only few of these inhibitors have demonstrated a significant blockade of STAT3 functions. Thus, identifying effective STAT3 inhibitor molecules that could revert the Sor resistance to develop individualized therapeutic strategies for clinical application in cancer is a need.

Among these promising molecules is Phloretin (PH). PH is a dihydrochalcone flavonoid (C_15_ H_14_ O_5_, Fig. 1b) that is mainly found in fruit, leaves, and roots of apple tree. In general, PH has high safety margin with less side effects. Several previous in vitro and in vivo studies showed that PH is not toxic to several non-cancerous cells such as epithelial breast cells MCF10A [18] and normal human dermal fibroblast [19]. It has also been reported that PH scavenges ONOO- and inhibits lipid peroxidation in rat liver microsomes [20]. Furthermore, pre- and post-treatment with PH significantly protected the liver from acetaminophen- and carbon tetrachloride (CCl4)-induced hepatotoxicity and reduced the degree of liver damage [20, 21]. Importantly, PH is shown to exhibit anticancer [22, 23], antitumor [24]^,^ and hepatoprotective effects with little side effects [25]. However, the exact mechanism of the antitumor effects of PH in HCC remains uninvestigated. Therefore, the main objectives of the current work were to a) explore the molecular mechanism(s) by which PH inhibits HCC proliferation in vitro and in vivo models and b) assess the role of PH in Sor-resistant xenografts. The results of the current work clearly showed that PH exhibited anticancer potential in vitro and retarded tumor growth via targeting the SHP-1/STAT3 and AKT/VEGFR2 signaling pathway.

## Methods

PH and Sor were purchased from Sigma-Aldrich (USA) and Selleck Chemicals LLC (Houston, TX). The SHP-1 inhibitor and PTP inhibitor III (CAS 29936-81-0) were purchased from Cayman Chemical (Ann Arbor, MI, USA). Smart-pool siRNA, including control (D-001810-10), SHP-1 (PTPN6) were all obtained from Dharmacon Inc. (Chicago, IL). The mutant SHP-1 constructs (DN1 and D61A) have been generated to mimic the open-form structure of SHP-1 as previously described [11]. Antibodies for immunoblotting such as p-STAT3, STAT3, survivin, p-Akt, Akt, p44/42 MAPK (Erk1/2) (Thr202/Tyr204), ERK, p-VEGFR2, VEGFR2, p- mTOR, mTOR, p-JAK2, JAK2, poly (ADP-ribose) polymerase (PARP) and cleaved caspase 3 were ordered from Cell Signaling Technology (Danvers, MA, USA). SHP-1, cyclin D1, Mcl-1, Ki67, b-actin antibodies were purchased from Abcam (Cambridge, MA, USA). BCA Protein Assay Kit was purchased from Pierce (Rockford, IL, USA).

### Cell Culture

Human hepatocellular carcinoma cells HepG2, SK-Hep1, Hep3B2.1-7, Huh-7, and PLC-5 were obtained from the American Type Culture Collection (Manassas, VA) and cultured in Dulbecco’s Modified Eagle's Medium (DMEM; GIBCO, MD, USA), containing 10% (v/v) fetal bovine serum (FBS; GIBCO, MD, USA) at 37 °C in a 5% CO_2_- humidified incubator. All cell lines were authenticated by STR profiling using the AmpFISTR Identifiler PCR amplification kit (Applied Biosystems, Foster City, CA).

### Establishment of Sor-resistant cells

Sorafenib-resistant cells HepG2^SR^ and Huh7^SR^ were established as described [26]. In brief, two Sor-resistant HCC cell lines (HepG2^SR^ and Huh7^SR^) were obtained by chronic exposure to Sor at low doses then increased to higher doses for a long period of time. Their ability of Sor resistance (SR) was further established and confirmed by incubating them with Sor at a starting concentration of 5 μM. Cells were continuously cultured with increasing concentrations of Sor by 1 μM per week for 1–2 months. The re-obtained SR-HCC cells were kept by culturing them in the presence of Sor.

### Patient samples

All pathologic samples of patients were obtained following written informed consent. The protocol conforms to the ethical guidelines of the 1975 Declaration of Helsinki as reflected in a priori approval by the institution's human research committee.

### Cell proliferation analysis

Cells were seeded in 96-well plate with 5,000 cells per well and were then treated with PH and/or Sor at indicated concentrations for 24, 48 and 72 h. After 24 h of incubation, 20 μl MTT (5 mg/ml) was added. The cultures were solubilized and the spectrophotometric absorbance was measured at 595 nm using a microtiter plate reader (Bio-Rad, USA). The number of viable cells was presented relative to untreated controls. The assay was repeated three times independently.

### Colony formation assay

Colony formation assay was conducted as described previously [14]. Briefly, HepG2^SR^ and Huh7^SR^ cells were seeded in 6-well plates (~1000-5000 cells per well) and treated with PH (50 μM). After incubation for 48 h, the cells were washed by PBS and then cultured in normal medium for two weeks. At the end of time point, cells were washed in PBS, fixed with 100% methanol and stained with a filtered solution of crystal violet (5% w/v). Colonies were visualized by Nikon Eclipse TS100 inverse microscope (Nikon Corporation, Japan) and pictures were captured using Nikon E8400 camera.

### DNA fragmentation

Cytoplasmic histone-associated DNA fragments were determined by the measurement of apoptotic cells using the Cell Death Detection ELISAPLUS Kit Roche (Indianapolis, IN) according to the manufacturer's instructions.

### Apoptosis assay

Approximately 2×10^5^ cells/well seeded in 6-well plates were treated with varying concentrations of PH for 48 h. Thereafter, the cells were collected and washed twice in ice-cold PBS before incubated with 5 μL FITC-Annexin V and 5 μL propidium iodide (PI) at room temperature for 15 min in the dark. Apoptotic cells were detected using an Annexin V-FITC Kit (BD Pharmingen, Franklin Lakes, NJ, USA) according to the manufacturer’s protocol. All samples were analyzed immediately using FACSCalibur flow cytometer (BD, San Jose, CA).

### Measurement of caspase-3, -8 and -9 activities

Caspase-3, -8 and -9 colorimetric Assay kits (BioVision, Inc.) were utilized as an additional method to evaluate apoptosis. The results are expressed as relative caspase-3, -8 and -9 activation in cells exposed to PH or Sor or both.

### HCC with ectopic STAT3 expression

STAT3 cDNA (KIAA1524) was purchased from Addgene plasmid repository (http://www.addgene.org). HepG2 and SK-Hep1 cells with ectopic expression of STAT3 derived from a single stable clone were exposed to PH. Briefly, following transfection, cells were cultured in the presence of G418 [27]**.** After eight weeks of selection, surviving colonies arising from stably transfected cells were selected and individually amplified.

### Gene knockdown using siRNA

siRNAs targeting STAT3 and AKT with scrambled control siRNAs were purchased from Dharmacon (Thermo Scientific, Chicago, IL, USA). Cells were transfected with siRNAs using Lipofectamine 2000 (Invitrogen Life Technologies, MD, USA) to knockdown gene expression as described previously [26].

### SHP-1 Phosphatase activity

SHP-1 activity was determined by using a RediPlate™ 96 Enzchek™ Tyrosine Phosphatase Assay Kit (Thermo Scientific, Chicago, IL, USA) according to the manufacturer's instructions.

### Phospho-STAT3 activity

Phospho-Stat3 (Tyr705) Sandwich ELISA Kit (Cell Signaling Technologies) was used to measure p-STAT3 activity according to the manufacturer's instructions.

### Western Blotting

Cells or the isolated independent tissues were lysed with RIPA Lysis Buffer (Santa Cruz Biotechnology, CA, USA) containing protease inhibitor (Roche Corp., Basal, Swiss) and phosphatase inhibitor (Roche Corp., Basal, Swiss) as described previously [28]. The proteins were separated by sodium dodecyl sulfate polyacrylamide gel electrophoresis (SDS–PAGE) and transferred to polyvinylidene difluoride (PVDF) membranes. The membranes were then incubated with primary antibodies overnight at 4°C followed by secondary antibodies for 1h at room temperature. Protein bands were visualized with enhanced chemiluminescence.

### Real-time reverse transcription PCR

Total RNA was extracted from tumors using TRIzol (Invitrogen) and RNeasy Mini Kit (Qiagen) and subsequently reverse-transcribed to cDNA using the SuperScript Kit (Invitrogen). Quantitative PCR (qPCR) was carried out on the MX3000P real-time PCR system (Stratagen, USA). The amplification specificity was confirmed by the melting curves. Relative mRNA levels were calculated based on the Ct values and normalized using β-actin expression, according to the equation: 2^−ΔCt^ [ΔCt = Ct _target gene_-Ct _β-actin_]. All experiments were performed in triplicate.

### Tumor xenografts

To investigate whether PH has a therapeutic effect on tumorigenesis in vivo, HepG2 (2 × 10^6^) and SK-Hep1 (3 × 10^6^) cells were injected SC inoculated into the posterior flank of nude mice and treated IP with PH 5 times a week for 3 weeks. Further to extrapolate our in vitro results to in vivo, we injected HepG2^SR^ (3 × 10^6^) and Huh7^SR^ (5 × 10^6^) cells in mice which received daily oral Sor at 10 mg/kg, which was used to maintain the Sor-resistant capacity of both HepG2^SR^ and Huh7^SR^ cells in mice [29]. When tumors became palpable ~100 mm^3^, animals were randomly divided and received PH, Sor or both. Tumor volume (TV) was calculated as follows: TV = (L × W^2^)/2 every third day till the animals were sacrificed and tumor weights were measured on day 30 after tumor excision. Survival rate was evaluated by the Kaplan–Meier method. Mice of each group were also monitored for other symptoms of side effects including food and water withdrawal and impaired posture or movement. At the termination of the experiment, the tumor tissues were harvested and used for immunohistochemistry. All procedures for animal experimentation used in the current study were approved by the Institutional Animal Ethics Committee, King Saud University, Riyadh, Saudi Arabia.

### Immunohistochemical staining

In brief, 5 μm-thick paraffin-embedded tumor sections from nude mice were stained for CD31, pSTAT3, Caspase-3, TUNEL and Ki67 using standard immunostaining protocols [30] and scanned at ×200 magnification. Six fields per section were analyzed and the expression levels of the target markers were semi-quantitatively assessed based on staining intensity by a board-certified pathologist.

### Molecular docking with SHP-1

SHP-1 was downloaded from RCSB PDB using pdb id: 3PS5. The protein was first prepared and the active sites were predicted using Discovery studio. his protein was then used for docking purpose using Autdock Vina [31–33]. Further, PH was docked with AKT1(4EJN). Each crystal structure of mentioned proteins was co-crystallized with a ligand bound at ATP binding site. Centre of mass of each bound ligand was calculated and utilized during molecular docking simulation. AutoDock vina program [31–33] was used for molecular docking and calculating the binding score of bound ligands of the proteins. Ligand efficacy (LE) of PH and other ligands was calculated as described by Hopkins et al [34]. UCSF Chimera and Ligplot programs were used for visualizing, editing and analysis of ligand and proteins interactions [35, 36].

### Statistics

Statistical analysis was performed with GraphPad Prism (GraphPad Software, San Diego, CA). Student’s *t* test was used to evaluate statistical significance of differences between two groups and *p* < 0.05 was considered statistically significant. Measurement values were expressed as mean ± SD.

## Results

### PH possesses anticancer and apoptotic effects in HCCs and potentiates Sor efficacy

To investigate the anticancer effects of PH against hepatic carcinoma, we first assessed growth inhibition in response to PH, in a panel of five HCC cell lines: SK-Hep1, Hep3B2.1-7, Huh7, PLC5, and one human hepatoblastoma cell line HepG2. Cell viability was determined by MTT assay after treatment for 24, 48 and 72 h. As shown in (Fig. 1c), PH significantly reduced cell viability in a concentration- and time-dependent manner. Next, we examined the apoptotic effect of PH on HCCs. PH alone significantly increased the activation of caspase-3, 8 and 9 in HepG2 and SK-Hep-1 cells as observed with colorimetric assay (Bio Vision, USA) (Additional file 1: FigureS1A). Importantly, inhibition of cellular growth and activation of apoptosis by PH were associated with a significant induction of DNA fragmentation in a concentration-dependent manner in all the five HCC cell lines (Fig. 1d). To further explore the mechanism of cell death mediated by PH in HCCs, we first measured the degree of cell apoptosis using Annexin V/PI staining. PH alone increased the percentage of HCC cells underwent apoptosis (Annexin V+/ PI+ population), a biomarker of apoptosis, in a concentration-dependent manner (Fig. 1e). Second, we studied the effect of PH on caspase-3 cleavage, where we found that PH induced the activation of caspase-3 cleavage and increased cleavage of poly (ADP- ribose) polymerase in all HCC cell line tested (Fig. 1f).

To answer the question of whether PH could enhance the cancer activity of Sor, we tested the effect of PH and Sor combination on cell proliferation and apoptosis in several HCC cells. Our results showed that Sor or PH alone decreased cell proliferation of HepG2, SK-Hep1, Hep3B2.1-7 cells in a concentration-dependent manner, whereas combination of Sor and PH significantly potentiated the suppression of the cell proliferation (Additional file 2: FigureS2A), while significantly increased the apoptosis as observed by Annexin V and DNA Fragmentation assays (Additional file 2: Figure S2B, S2C). These data not only indicate that PH inhibits HCC cell proliferation through induction of apoptosis but also shed the light on its synergistic effect on the antitumor activity of Sor.

To explore the role of STAT3 and SPH-1 in the anticancer activity of PH, two independent experiments were conducted. First, we measured the effect of combination therapy on p-STAT3 and SHP-1 activities. Our results showed that PH in combination with Sor markedly decreased the p-STAT3 (Additional file 2: Figure S2D, S2E) while enhanced SHP-1 (Additional file 2: Figure S2F) activities as compared to monotherapy. Second, we next examined whether knockdown of STAT3 and Akt by siRNAs could potentiate the anticancer activities of PH in HCC cells. For this purpose, HepG2 and SK-Hep1 cells were transfected with control, STAT3 siRNA or Akt siRNA for 24 h and then further combined for 48 h with PH (Additional file 3: Figure S3A, S3B). Our results show that knockdown of both STAT3 and Akt by siRNA induced apoptosis in both cells, suggesting that PH inhibits Sor-induced STAT3 and Akt activation, thus reversing Sor resistance in HCCs.

### PH induced HCC cell death via inhibiting STAT3/AKT/ERK signaling

**Fig. 2:**
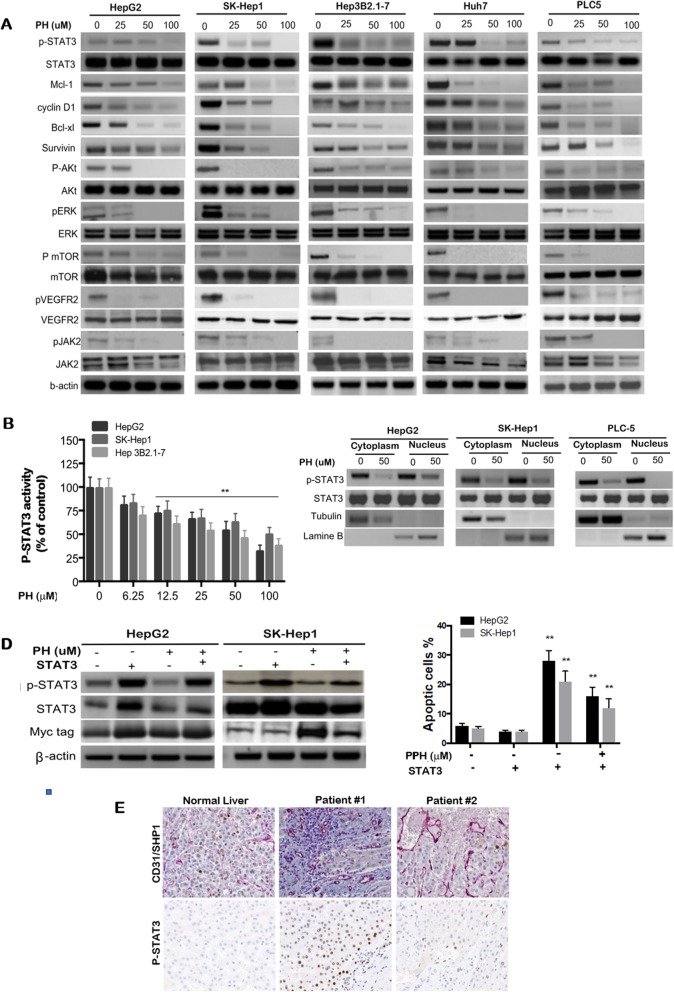
PH inhibits STAT3, AKT/mTOR and RAS/MAPK signaling in HCCs. **a** Cells were treated for 48h with indicated concentrations of PH and phosphorylated and total protein levels of the target proteins were evaluated by Western blotting. **b** pSTAT-3 activity was measured by ELISA (**c**) PH inhibits cytoplasmic, nuclear STAT3 phosphorylation in HCCs at 50uM. **d** Cells (wild-type or ectopic expression of STAT3) were exposed to PH at 50uM for 24 hours. Percentage of apoptotic cells was analyzed by flow cytometry. **e** Representative images from two patients with double immunostaing of CD31/SHP1 and pSTAT3. Experiments were conducted in triplicate and mean values ± SD (bars) are shown. *p<0.05, and **p<0.01 compared to control

In order to study the function of signal transduction cascade in hepatocarcinogenesis, we investigated the effect of PH on the phosphorylation of STAT3, MAPK and the expression of Akt and JAK2 proteins. The results illustrated in Fig. 2a shows that PH significantly inhibited STAT3 protein phosphorylation at tyrosine 705 transactivation of STAT3 and its downstream pro-proliferative proteins Mcl-1, cyclin D1 Bcl-xl and Survivin in a concentration-dependent manner. Furthermore, although PH did not affect the total STAT3 activity levels, it significantly inhibited p-STAT3 activity levels in a concentration-dependent manner (Fig. 2b) and its protein expression in both the cytoplasm and nucleus (Fig. 2c). In addition, PH downregulated the expression of p-Akt, p-ERK, p-mTOR, p-VEGFR2 and p-JNK in a concentration-dependent manner without having effect on the total expression of these proteins (Fig. 2a).

To further validate the role of STAT3 in PH-induced apoptosis, we generated stable STAT3-overexpressing HepG2 and SK-Hep1 and tested the molecular changes induced by PH treatment. Ectopic expression of STAT3 increased p-STAT3 protein expression (Fig. 2d) and decreased the percentage of apoptotic cells (Fig. 2d), suggesting that activated STAT3 signaling counteracts the apoptotic activity of PH in HCCs. To tested whether these in vitro results are extrapolated into human model, we performed immunohistochemistry (IHC) assay to examine the levels of CD31, SHP1 and pSTAT3 in liver tissues obtained from healthy and HCC patients. IHC staining shows strong SHP-1 correlation with p-STAT3 expression in normal liver and vice versa in human tumor samples (Fig. 2e).

### PH increased SHP-1 phosphatase activity by direct interaction

**Fig. 3:**
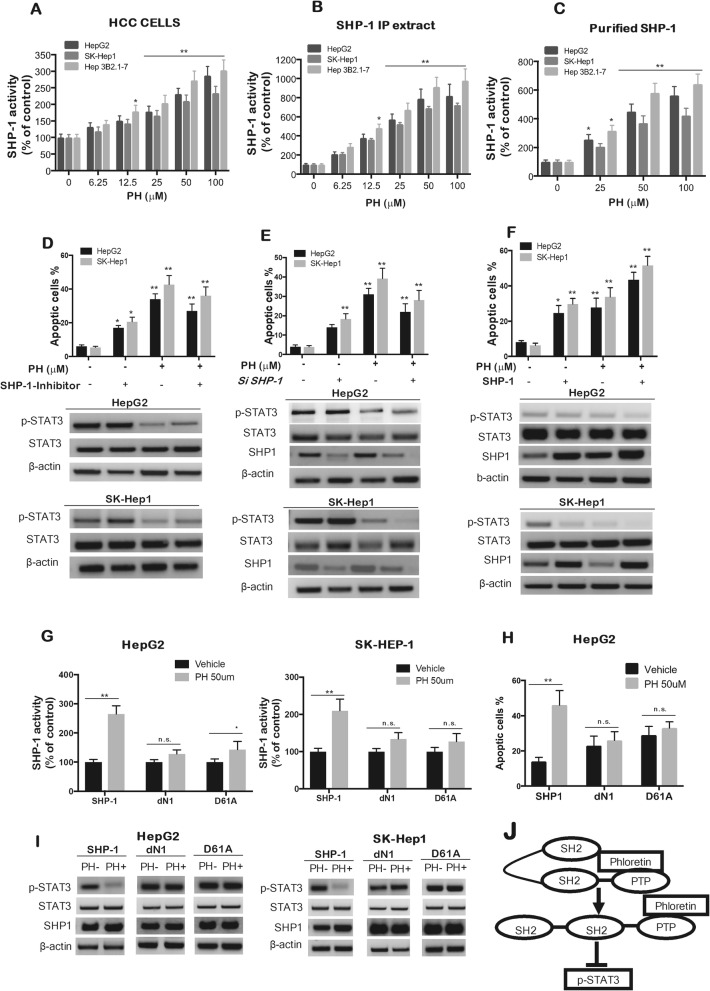
PH enhances SHP-1 tyrosine phosphatase activity. **a** SHP-1 phosphatase activity. **b** The SHP-1-containing lysates of HCCs were collected by SHP-1 antibody and incubated with PH for 30 min. **c** PH enhanced SHP-1 activity in cell-free SHP-1 protein. **d** HCCcells were pre-treated with 25 μM specific SHP-1 inhibitor (PTPIII) for 30 min and then co-incubated with PH 50 μM for additional 48 h. **e** Knockdown of SHP-1 reversed the biological effects of PH on p-STAT3 and apoptosis. **f** PH reinforced apoptosis in stably expressing SHP-1 HCCs. **g** Purified dN1 and D61A mutants of SHP-1 were insensitive to PH treatment. **h** Percentage of apoptotic cells was analyzed by flow cytometry. **i** Effect of dN1 and D61A mutants on STAT3 expression. **j** Molecular model of PH. Experiments were conducted in triplicate and mean values ± SD (bars) are shown. *p < 0.05, and **p < 0.01 compared to control

SHP-1 activity has a critical role in induction of apoptosis in different cancer types [11–15]. To test whether SHP-1 is involved in PH-induced STAT3 suppression, we first tested the effect of PH on SHP-1 phosphatase activity. PH alone significantly enhanced the phosphatase activity of SHP-1 in a concentration-dependent manner in all tested HCC cell lines (Fig. 3a). Thereafter, we studied the effect of PH on SHP-1–containing cell lysates. In brief, HepG2, SK-Hep-1 and Hep3B2.1-7 cells were immunoprecipitated with anti-SHP-1 antibody, then the protein extracts containing SHP-1 complex was further incubated with different PH concentrations for 30 min before SHP-1 activity was determined. As shown in Fig. 3b, PH (50 μM) significantly increased the phosphatase activity of SHP-1–containing lysates in HepG2, SK-Hep-1 and Hep 3B2.1-7 by approximately 7.89, 6.91 and 9.78-fold, respectively. For more specific identification, we incubated pure SHP-1 recombinant protein for 30 min with increasing concentrations of PH (25-100 μM) followed by measurement of the SHP-1 phosphatase activity. Our results showed that PH (50 μM) treatment increased the SHP-1 phosphatase activity in HepG2, SK-Hep1and Hep3B2.1-7 cells by approximately 4.5-, 3.5- and 5.8-fold, respectively, suggesting that PH potentially acts on SHP-1 by direct interaction (Fig. 3c).

To further validate the role of SHP-1 in PH-mediated molecular events and apoptosis, we tested whether inhibition of SHP-1 either chemically using a specific inhibitor (PTP III) or genetically via transient transfection of SHP-1-targeted siRNAs would alter PH effects in HCC cells. Our results showed that blocking of SHP-1 either using PTP III (Fig. 3d) or siRNA (Fig. 3e) abolished PH-mediated cell death and downregulation of p-STAT3 in both HepG2 and SK-Hep1 cells. PH induced significant cell death and inhibition of STAT3 in HepG2, SK-Hep-1 and Hep3B2.1-7 cells overexpressing SHP-1 (Fig. 3f). This data suggests that PH increases SHP-1 activity by direct interaction that subsequently results in SHP-1–mediated inhibition of p-STAT3.

### PH interfered with inhibitory N-SH2 domain and relieved autoinhibition of SHP-1

To investigate the mechanism through which PH increased SHP-1 tyrosine phosphatase activity, we transfected HepG2 and SK-Hep1 cells with wild-type or mutant SHP-1 (dN1 and D61A), thereafter we examined the effect of PH on SHP-1. The intramolecular inhibition of SHP-1 is protected by various biochemical associations between N1 and PTP catalytic domain, such as Asp61 and Lys362 (salt bridge) [37]. Deletion of the autoinhibitory N-SH2 domain (dN1) and single-mutant D61A of SHP-1 was used to mimic the open-form structure of SHP-1. PH co-incubated with purified wild-type or mutant SHP-1 proteins in vitro enhanced the activity of wild-type SHP-1 protein to nearly 2.6 and 2-folds in HepG2 and SK-Hep1 cells, respectively (Fig. 3g). As compared to Sor, sc-43 and sc-60, PH increased the phosphatase activity in recombinant SHP-1 proteins expressing dN1 or D61A mutants in both HepG2 and SK- Hep1 cells. In addition, wild-type SHP-1–transfected HCC cells showed a marked decrease in p-STAT3^Tyr705^ level (Fig. 3i) and a significant increase in apoptosis (Fig. 3h, Additional file 2: Figure S2G). Whereas no such effect was observed in dN1 or D61A mutant SHP-1–transfected HCCs (Fig. 3i). These findings suggest that PH activates SHP-1 by disruption of autoinhibition of SHP-1, leading to reduced p-STAT3^Tyr705^ level and eventual induction of apoptosis in HCC cells (Fig. 3j). Thus, SHP-1 targeting is critical in PH-induced anti-HCC activity.

### PH overcome Sor resistance in HCCs

Sor-resistant cell lines HepG2^SR^ and Huh7^SR^ were used to study whether PH could overcome the resistance in HCC cell lines and whether p-STAT3 and SHP-1 are involved. Resistance of HepG2^SR^ and Huh7^SR^ cells to Sor has been confirmed by the significant decrease in growth inhibition (Additional file 4: Figure S4A), no significant changes on p-STAT3 protein expression and percentage of apoptotic cells (Additional file 4: Figure S4B), no induction of DNA fragmentation in both resistant cells (Additional file 4: Figure S4C). These results clearly suggest that Sor-resistant cells become refractory to Sor-induced apoptosis.

**Fig. 4:**
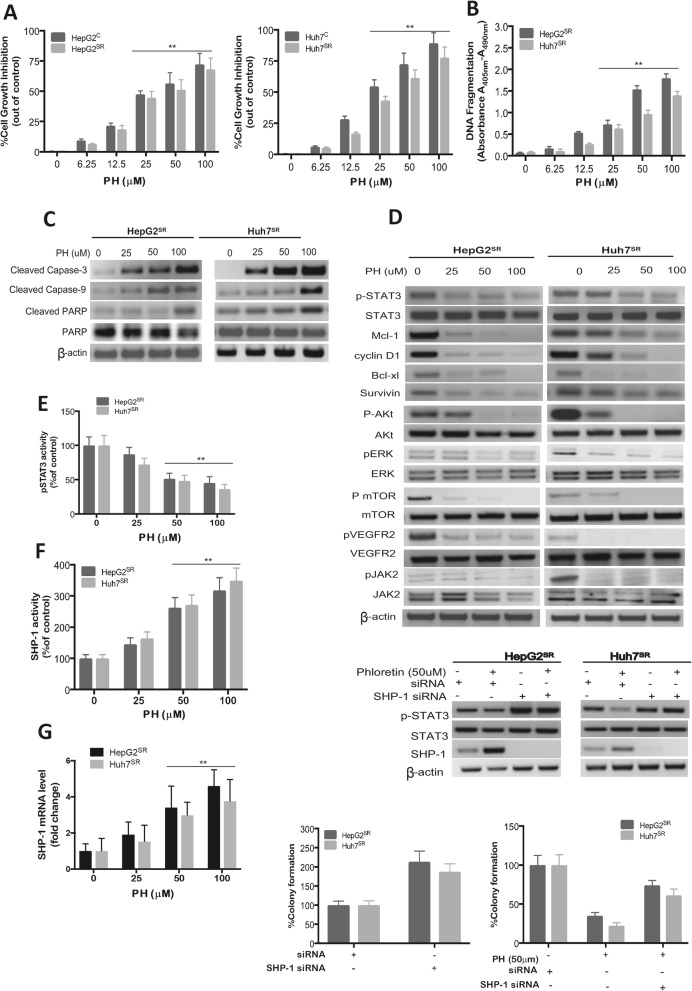
PH overcomes Sor resistance in HepG2^SR^ and Huh7^SR^ cells in vitro. **a** PH inhibited cell growth in Sor resistant HepG2^SR^ and Huh7^SR^ cells. Cell viability was assessed by MTT assay. **b** PH enhanced DNA fragmentation in SR-HCCs. **c** The protein levels of caspase-3, caspase-9 and PARP after exposing SR-HCCs to PH. **d** PH inhibited STAT3, ERK, AKT/mTOR signaling in HCC cells. **e** pSTAT-3 activity was measured by ELISA. **f** SHP-1 phosphatase activity in HepG2^SR^ and Huh7^SR^. **g** SHP-1 mRNA levels were analyzed by qPCR. **h** SR-HCC cells were transfected with control siRNA or SHP-1 siRNA for 48 h there exposed to PH and subjected to Western blot assay of p-STAT3 and SHP-1 or seeded on a 6-well plate for the colony forming assay. Experiments were conducted in triplicate and mean values ± SD (bars) are shown. *p < 0.05, and **p < 0.01 compared to control

We next explored the effect of PH in Sor-resistant cells. The results illustrated in Fig. 4 shows that PH attenuated cell growth of both HepG2^SR^ and Huh7^SR^ cells (Fig. 4a) and induced DNA fragmentation in both cells (Fig. 4b). This was further supported by the ability of PH to significantly inhibit cell growth as measured by colonogenic assay (Additional file 4: Figure S4D) and by increased the protein expressions of apoptotic genes, such as cleaved caspase-3, -9 and PARP (Fig. 4c). Taken together, these results suggest that PH has potent inhibitory effect on Sor-resistance HCCs.

### PH overcome STAT3-, AKT-, MAPK- and VEGFR2-dependent resistance to Sor in HCCs

To further explore the mechanisms of PH effect on Sor-resistant HCC cells, we investigated the effect of PH on the protein expression and activity of p-STAT3 and SHP-1 in Sor-resistant HepG2^SR^ and Huh7^SR^ cells. PH treatment resulted in downregulation of p-STAT3 protein expression and its targets in a concentration-dependent manner (Fig. 4d) with significant decrease in p-STAT3 activity at 50 and 100 μM concentrations using ELISA (Fig. 4e). With regards to SHP-1, PH treatment caused marked increases in SHP-1 activitiy (Fig. 4f) and mRNA (Fig. 4g) levels in both HepG2^SR^ and Huh7^SR^ cells.

STAT3 has been reported to be activated by JAK2 [38], therefore, we test the effect of PH on JAK2. Our results showed that PH inhibited phosphorylation of JAK2 in a concentration-dependent manner in Sor-resistant cells (Fig. 4d). Previous study has shown that Sor-induced Akt, ERK and VEGFR2 activation has been reported in both Sor-resistant and parental HCC cells [3, 4, 11] and that increased p-Akt, p-ERK and p-VEGFR2 expression is responsible for resistance to Sor. We next examined alterations of the key molecules in the Akt/mTOR, MAPK and VEGFR2 pathways, pathways that have been shown to be responsible for resistance to Sor [11]. Our results showed that Sor-resistant cells expressed higher levels of p- Akt, p-ERK and p-VEGFR2 resulting in upregulation of p-mTOR (Fig. 4d). Importantly, PH treatment downregulated p-Akt, p-ERK, p-VEGFR2 and p-mTOR protein expression in a concentration-dependent manner but did not affect the total protein expression (Fig. 4d).

To further investigate the mechanism through which PH increased SHP-1 and decreased p-STAT3 in Sor-resistant cells, we knocked down SHP-1 using siRNA to address whether SHP-1 is mediating Sor resistance. As shown in Fig. 4h, while PH treatment increased SHP-1 and repressed p-STAT3 proteins with inhibition of colony formation, SHP-1 silencing abolished the inhibitory effects of PH on p-STAT3 and colony formation in Sor-resistant cells. These results not only indicate the critical role of SHP-1 in PH-induced inhibition of cell growth, but also suggest that targeting SHP-1 by PH could overcome Sor resistance.

### PH combination with Sor synergizes the antitumor effect in SR-HCCs

**Fig. 5:**
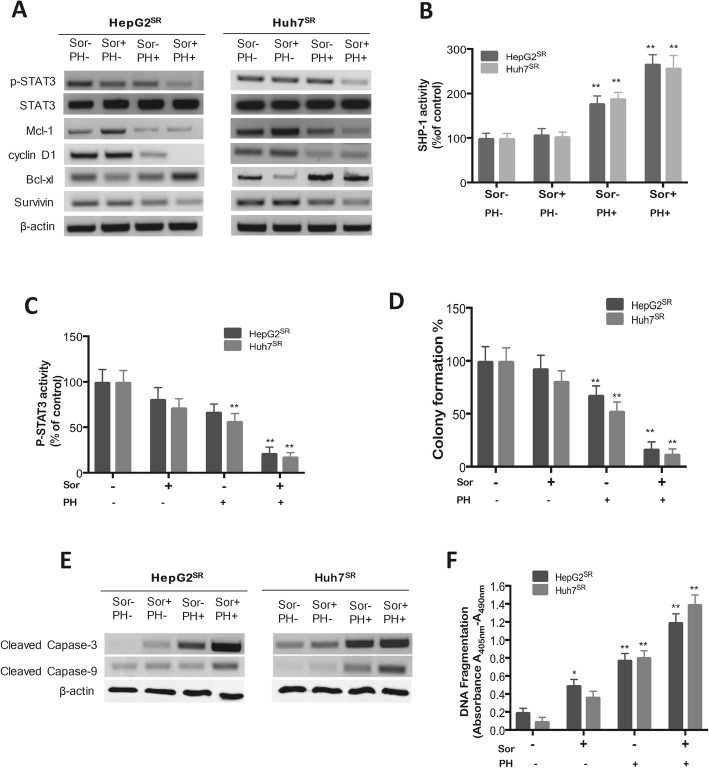
PH potentiates the effect of Sor in HepG2^SR^ and Huh7^SR^ cells in vitro. **a** Effect of PH (50 μM), Sor (10 μM) or both on p-STAT3 and its downstream proteins in SR-HCCs. **b** SHP-1 phosphatase activity in SR-HCCs exposed to PH, Sor or both for 48 h. **c** pSTAT3 activity was measured by ELISA. **d** Colony formation assay. **e** The protein levels of cleaved caspase-3 and -9 were determined by Western blot assay (**f**) DNA fragmentation assay for cells exposed to PH, Sor or both for 48 h. Experiments were conducted in triplicate and mean values ± SD (bars) are shown. *p < 0.05, and **p < 0.01 compared to control

To know whether PH could potentiate the antitumor efficacy of Sor, we studied the combined effect of PH and Sor in Sor-resistant HepG2^SR^ and Huh7^SR^ cells. Combined therapy resulted in significant downregulation of p-STAT3 and its downstream signal proteins in both Sor-resistant cell lines (Fig. 5a). We further determined the effect of combination therapy on SHP-1 and p-STAT3 activity in target gene regulation. Fig. 5b shows that PH alone increased SHP-1 activity, while further potentiated SHP-1 activity in the presence of Sor. In addition, combination therapy further downregulated p-STAT3 activity (Fig. 5c), further inhibited colony forming ability in both Sor-resistant cells (Fig. 5d), and significantly increased cleaved caspase-3 and 9 proteins (Fig. 5e) and DNA Fragmentation (Fig. 5f). Taken together, these results strongly suggest that PH potentiates the antitumor efficacy Sor.

### PH inhibited tumor growth via a SHP-1/STAT3-related signaling pathway in vivo

**Fig. 6:**
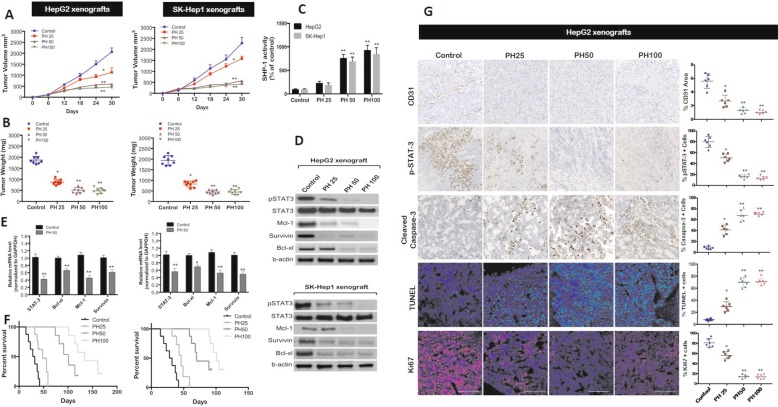
PH inhibits tumor growth via downregulating STAT3-related signaling pathway. **a** PH significantly inhibited tumor growth in HepG2 and SK-Hep1 tumor-bearing mice. **b** Tumor weight. **c** The in vivo SHP-1 phosphatase activity. **d** The protein levels of p-STAT3 and its downstream proteins were determined by Western blot assay. **e** STAT3 and related mRNA levels were analyzed by qPCR. **f** Kaplan–Meier plot showing animal survival after treatment with PH at indicated doses (*n* = 8). **g** Immunohistochemical staining (Left panel) of CD31, pSTAT3, cleaved caspase-3, Ki67 and TUNEL in tumor xenografts. Magnification: ×200. Data represents mean values from five random fields per tumor section. Scale bar, 50 μm. Mean values ± SD (bars) are shown. *p < 0.05, and **p < 0.01 compared to control

To investigate whether PH has anti-tumorigenic effect in vivo, we subcutaneously injected HepG2 (2 × 10^6^) and SK-Hep1 (3 × 10^6^) cells into the posterior flank of nude mice followed by IP injection of PH at indicated doses (25, 50, and 100 mg/kg) 5 times a week for 3 weeks. Tumor growth was measured every 6 days till the animals were sacrificed on the 30^th^ day. Our results showed that PH decreased tumor volume (Fig. 6a) and tumor weight (Fig. 6b) in both HepG2 and SK-Hep1 xenografts in a dose-dependent manner. Maximal efficacy was demonstrated with both 50 and 100 mg/kg dose of PH. To further understand the underlying mechanism in close proximity of hepatic cancer, we analyzed the levels of p-STAT3 protein expression and phosphatase activity of SHP-1. PH treatment significantly increased the SHP-1 tyrosine phosphatase activity (Fig. 6c), whereas inhibited the expression of p-STAT3^Tyr705^ and its downstream signaling targets (Mcl-1, survivin, Bcl-xl) both at the protein (Fig. 6d), and mRNA (Fig. 6e) levels. All together, these effects were associated with significant survival benefits by PH compared with mice treated with vehicle in both HepG2 and SK-Hep1 xenograft (Fig. 6f). The results of the in vivo study suggest that suppression of tumorigenicity by PH in vivo is due to its enhancement of SHP-1 activity that directly targeted p-STAT3^Tyr705^ expression.

Angiogenesis plays important role in tumor growth, progression and metastasis [39]. Therefore, we examined whether PH inhibits angiogenesis in vivo. Immunostaining with CD31 markedly decreased angiogenesis in tumor tissues in both HepG2 (Fig. 6g) and SK-Hep1 (Additional file 5: Figure S5A) xenografts treated with PH in a dose-dependent manner. Significant decrease in p-STAT3 expression in both HepG2 (Fig. 6g) and SK-Hep1 (Additional file 5: Figure S5F) xenografts was observed. TUNEL labelling and caspase-3 expression revealed a high induction of apoptosis by a factor of 9 and 10 in HepG2 (Fig. 6g) and 8 and 11 in SK-Hep1 (Additional file 5: Figure S5F) with PH 50 mg/kg. There was a significant decrease in Ki67+ cells in PH treated tumors (Fig. 6g; Additional file 5: Figure S5F). These results suggest that PH inhibits tumor growth by impairing angiogenesis, cell proliferation and inducing apoptosis in both HepG2 and SK-Hep1 tumors.

Further, we tested the combined effect of PH and Sor on the tumor volume in both HepG2 and SK-Hep1 xenografts. PH (50 mg/kg) alone decreased the tumor volume by 75% and 71%, whereas Sor alone (20 mg/kg) decreased the tumor volume by 54% and 51% in HepG2 and SK-Hep1 xenografts, respectively (Additional file 5: Figure S5A). Importantly, combination therapy of PH and Sor produced an additive decrease in tumor volume by 83% and 89% in both cells, respectively (Additional file 5: Figure S5A). However, no significant changes in body weight loss or other clinical signs of toxicity were observed in any groups (Additional file 5: Figure S5B). Further we examined whether the potentiation effect of combined therapy of PH and Sor on tumor volume is associated with synergistic effect on SHP-1 and p-STAT3. Additional file 5: Figure S5D shows that a synergistic increase in SHP-1 activity with a further decrease in p-STAT3 activity and downstream proteins as compared to monotherapy. No histological differences were observed in various organs between the control and PH treated groups (Additional file 5: Figure S5C). These results suggest that PH acts as a potent STAT3 inhibitor and SHP-1 enhancer, and thus induces its anti–hepatocellular carcinoma effect via a STAT3-related signaling pathway.

### PH possessed antitumor effect against Sor-resistant xenograft tumors

**Fig. 7:**
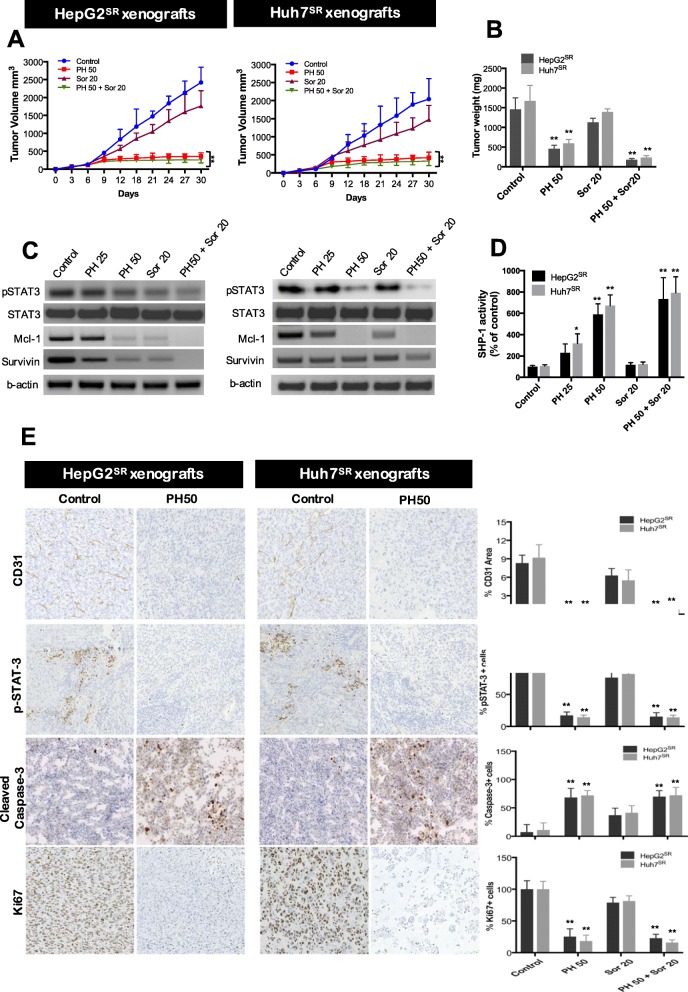
PH overcomes the Sor resistance in HepG2^SR^ and Huh7^SR^ xenografts in vivo. **a** PH significantly reduced tumor growth in SR-HCCs xenografts. When tumors reached 100 mm^3^, the mice were treated with PH, Sor or both and then tumor growth was measured every 3 days. **b** Tumor weight was measured on day 30 after tumor excision. **c** The protein levels of p-STAT3 and its downstream proteins were determined by Western blot. **d** The in vivo SHP-1 phosphatase activity. **e** Immunohistochemical staining of CD31, p-STAT3, cleaved caspase-3, Ki67 and TUNEL in HepG2^SR^ and Huh7^SR^ xenografts. Magnification: ×200. Data represents mean values from five random fields per tumor section. Scale bar, 50 μm. Mean values ± SD (bars) are shown. *p < 0.05, and **p < 0.01 compared to control

To assess the antitumor activity of PH against Sor-resistant xenografts, we injected HepG2^SR^ (3 × 10^6^) and Huh7^SR^ cells (5 × 10^6^) cells in mice which have received daily oral Sor dose (10 mg/kg), a dose that is used to maintain the Sor-resistant capacity of both HepG2^SR^ and Huh7^SR^ cells in mice [15]. When tumors became palpable ~100 mm^3^, animals were randomly divided in four groups as follows: first group received only vehicle (control), second group received PH (50 mg/kg), third group received Sor (20 mg/kg), fourth group received both PH plus Sor. As demonstrated in Fig. 7, SR-HCC tumors were shown to be resistant to Sor in vivo since there was a slight decrease in tumor volume and weight as compared to vehicle treated group. However, PH treatment decreased tumor volume and weight by 73% and 68% in HepG2^SR^ and by 74% and 64% in Huh7^SR^ cells. Treatment of mice with combination therapy of PH plus Sor produced additive effect by decreasing tumor volume by 83% and 88% in HepG2^SR^ and 80% and 86% in Huh7^SR^ xenografts (Fig. 7a and b).

We further investigated the effect of PH, Sor or combination treatment on SPH-1 and p-STAT3 expression in HepG2^SR^ and Huh7^SR^ xenografts. In consistency with the in vitro and in vivo results, Western blot assay results showed a significant decrease in p-STAT3 protein expression and its downstream targets (Fig. 7c) and an increase in SHP-1 activity (Fig. 7d). In addition, immunostainings for CD31, p-STAT3, caspase-3 and ki67 showed that Sor had a weak inhibitory effect on cell proliferation with weak pro-apoptotic activity against Sor-resistant tumors (Fig. 7e), whereas, PH significantly decreased CD31, p-STAT3 and Ki67 expression while induced caspase-3 activity in both HepG2^SR^ and Huh7^SR^ xenografts. These Inhibitory effects were also observed in combination with Sor but is not significant as compared to PH alone. Together, our data indicate that PH inhibited the tumor growth both in vitro and in vivo through a STAT3 inhibition mechanism involving SHP-1.

### Molecular docking

**Fig. 8:**
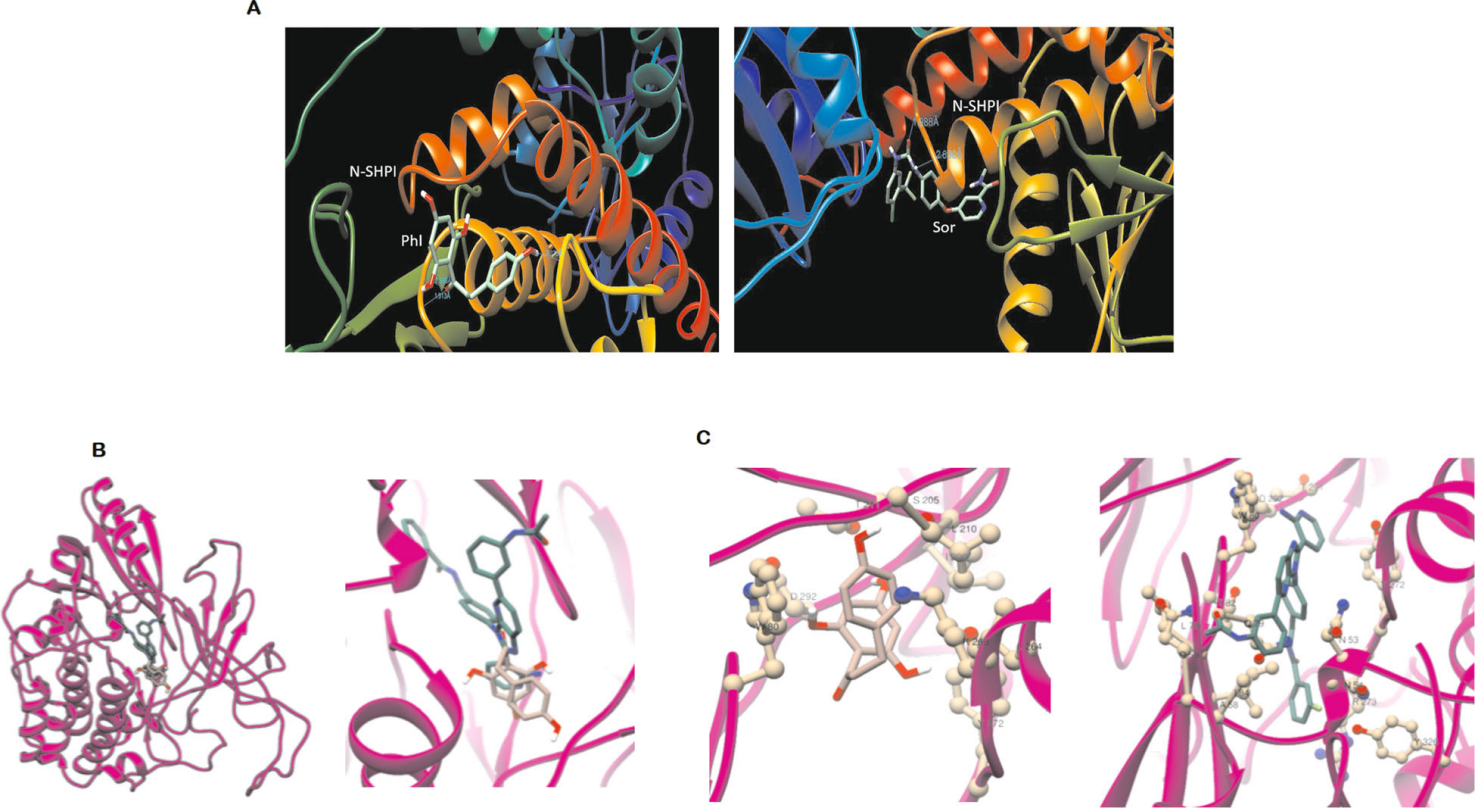
Molecular Docking. **a** PH shows 3 BS of protein with 3H-bond, Sor shows binding near to active site (with 2-H bond). **b** N-Sh2, C-Sh2, and PTP domains of Shp1 protein (2B3O) structure. The four binding sites (BS) identified from Swiss dock models are marked in the imaged along with the predicted binding affinities for Sor (SFB) and PH (PHL). **c** Docking of PH in ATP binding site of AKT1 protein (PDB id: 4EJN). PH and 0R4 are in rosy brown and sea green respectively (Left)

We performed docking of these ligands with SHP-1 (pdb id: 3PS5) individually, using Autodock Vina (Fig. 8a). PH docks within the predicted active site (Ser 453 to Thr 460) into the interface of N-SH2 and PTP domain and forms 3H-bonds within the active site with docking score of -6.5 (Fig. 8a). Sor docks in a region spanning from Asp 419 to Ser 474 and forms 2H bonds with docking score of -7.9. These results showed that PH is equally potential candidate as Sor. All molecules have bound at AKT1’s ATP binding site, whose coulombic charge distribution surface is positively charged around opening but hydrophobic in deeper side (Fig. 8b, c). This charge distribution plays a role in binding of molecules if they are physicochemical complementary with interacting surface. The binding affinity and ligand efficiency scores of 0R4, an inhibitor of AKT1 (PDB id: 4EJN) are -13.9kcal/mol and -0.32kcal/mol respectively. The binding energy of PH is -7.7Kcal/mol and its ligand efficiency is -0.39 kcal due to presence of 20 heavy atoms only. This analysis shows that PH binds more efficiently with than 0åR4.

## Discussion

Over-expression and hyperactivation of STAT3 has been reported in various human tumors. STAT3 and MAPK play an important role in signal transduction cascades, which take part in cellular physiological growth, development, mitogenesis and differentiation, and cellular malignant transformation [17]. One of the novel strategies to prevent sustained STAT3 over-expression in cancer cells is through the activation of intrinsic regulators such as SHP-1 [40]. This effect is supported by the findings of several studies revealed that loss of SHP-1 leads to constitutive activation of STAT3. In addition, SHP-1 has been shown to dephosphorylate STAT3 directly to silence the JAK/STAT pathway [38]. The current study provides the first evidence that PH demonstrates a significant anti-proliferative effect in HCCs via STAT3 pathway inhibition. Our data suggest that PH inhibits the JAK/STAT3 signaling pathway by stimulating Tyr^705^ dephosphorylation of STAT3 and inhibiting p-JAK2. PH also upregulates SHP-1 activity and induces SHP-1-dependent p-STAT3 downregulation.

Activation of the AKT/mTOR and RAS/MAPK cascades is frequently observed and associated with aggressive tumor phenotypes and poor prognosis in human HCC [39]. SHP-1 is a negative regulator of the cell cycle, as well as inflammatory and JAK/STAT pathways in cancer progression [41]. We have observed that PH significantly decreased expression of p-ERK p-Akt and p-mTOR in all tested HCCs. Inhibiting SHP-1 using specific inhibitor, PTP inhibitor III, upregulates p-STAT3 and rescues the SC-43-induced apoptosis. The antiproliferative activity of PH was significantly counteracted by SHP-1 knockdown using siRNA suggesting that PH mainly targets SHP-1 and downregulates p-STAT3, and thus inhibits cell proliferation and induces apoptosis. The dN1 mutant showed slight increase in SHP-1 activity in both HCCs, but it was not significant, whereas D61A mutants demonstrated a significant increase in SHP-1 activity only in HepG2 not in SK-Hep1 indicating that D61 site of the inhibitory N-SH2 domain is crucial for PH-induced SHP-1 upregulation, although it behaves differently in HepG2 compared to SK-Hep1 cells. This could be explained by its flexible orientation and unknown mechanism for searching of phospho-tyrosine activators [11]. PH-induced downregulation of p-STAT3 was found in HCCs cells expressing wild-type (WT) of SHP-1. However, ectopic expression of dN1 and D61A restored the expression of p-STAT3. Together, the data suggest that PH may affect SHP-1 by switching the confirmation from autoinhibitory (closed) to active (open). Our in vivo studies recaptured our findings in vitro, that PH showed significant antitumor activity via inhibition of p-STAT3 and upregulation of SHP-1 activity in HCC- xenografts and improved the overall survival.

**Fig. 9:**
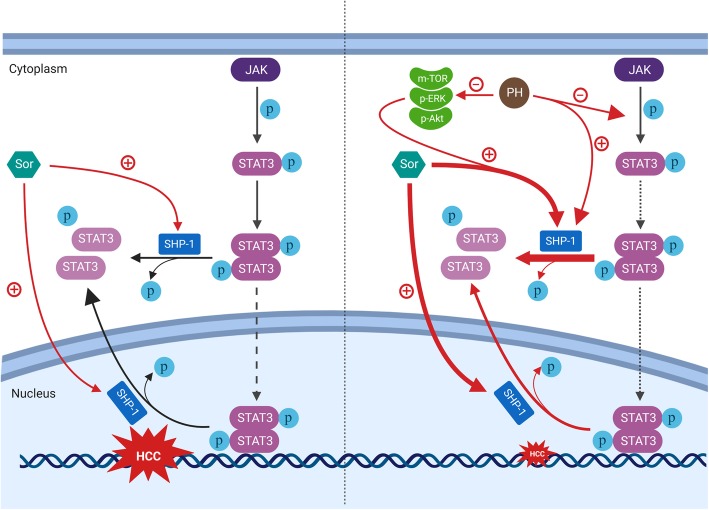
Graphical abstract for the proposed mechanism of action of PH in HCC

Several mechanisms are involved in the acquired resistance to Sor, such as crosstalk involving PI3K/Akt and JAK-STAT pathways, the activation of hypoxia-inducible pathways and epithelial-mesenchymal transition [42, 43]. Therefore, it is of great impact to develop individualized therapeutic strategies for treatment of Sor resistance cases. Since our current observations clearly show that PH activated SHP-1 and reduced the level of p-STAT3 activity and expression in vitro and in vivo, we hypothesized that PH overcomes STAT3-dependent Sor-resistance in HCC cells. This hypothesis was evidenced in two Sor-resistant HCC cell lines by which PH decreased cell viability, colony formation and increased apoptosis. Sor-resistant HCC cells express higher levels of pAkt, m-TOR, pERK, p-STAT3, p-JAK2, but lower levels of SHP-1 and p-SHP-1, indicating that the JAK-STAT pathway participates in the acquired resistance to Sor in HCC. PH overcomes the Sor resistance via downregulating the expression of pAkt, pERK, p-STAT3, p-JAK2 along with decrease in pVEGFR2 in SR-HCC. In addition, we confirmed the anticancer activity of PH to overcome Sor resistance of HCC in experimental animals. We inoculated SR-HCCs to animals and found that PH significantly retarded the growth of tumors in SR-HCC xenografts. We also found that PH used in combination with Sor displayed synergistic effect in vitro in SR-HCC but not in vivo tumors. To our knowledge, this is the first report showing that PH inhibits HCC in vitro and in vivo via targeting STAT3/SHP1signaling pathway. A proposed mechanism of action of PH on Sor-resistant and -sensitive HCC is illustrated in Fig. 9.

## Conclusion

In summary, our study demonstrated the preclinical activity of PH, which inhibits hepatic carcinoma in vitro and in vivo and prolonged mouse survival via molecular targeting of STAT3/Akt/mTOR/JAK2/VEGF2 pathways. PH holds promise as an adjuvant drug for HCC treatment and is equally as potential as Sor. This may represent an alternative strategy in the treatment of HCC.

## Additional files

Additional file 1: **Figure S1.** Colorimetric assay of caspase-3, -8 and -9 activity in HepG2 and Sk-Hep1. (A) Cells were treated with PH or Sor or both at indicated doses for 48 h and caspase-3, -8 and -9 activities were measured by colorimetric kits from Abcam, Cambridge, MA, USA). Experiments were conducted in triplicate and mean values ± SD (bars) are shown. *p < 0.05, and **p < 0.01 versus control. (DOCX 161 kb)

Additional file 2: **Figure S2.** PH potentiates the effect of Sor in vitro in HCCs. (A) HepG2, SK-Hep1 and Hep 3B2. 1-7 cells were treated with PH or Sor or both at indicated doses for 48 h and growth inhibition was assessed by MTT assay. (B) PH enhanced DNA fragmentation. PH or Sor or both at indicated doses for 48 h and DNA fragmentation was measured by cell death detection ELISA. (C) Apoptosis was analyzed by annexin V labeling and FACS analysis. Annexin V (+) cells were quantified. (D) pSTAT-3 activity was measured by ELISA. (E) The protein levels of p-STAT3 was determined by western blot (F) HCC cells were exposed PH or Sor or both at the indicated doses for 48 h and SHP- 1 phosphatase activity was determined. (G) Purified dN1 and D61A mutants of SHP-1 were insensitive to PH treatment. (H) Percentage of apoptotic cells was analyzed by flow cytometry in purified dN1 and D61A mutants of SHP-1 treated with PH. Experiments were conducted in triplicate and mean values ± SD (bars) are shown. *p<0.05, and **p<0.01 versus control. (DOCX 161 kb)

Additional file 3: **Figure S3.** Knockdown of STAT3 and Akt sensitizes PH-induced apoptosis. (A) siRNA against STAT3 enhanced apoptosis in HepG2 and SK-Hep1 cells. (B) siRNA against Akt enhanced apoptosis in HepG2 and SK-Hep1 cells. Experiments were conducted in triplicate and mean values ± SD (bars) are shown. *p<0.05, and **p<0.01 versus control. (DOCX 71 kb)

Additional file 4: **Figure S4.** Sor-resistant HCC cells are insensitive to Sor. (A) HepG2, HepG2-SR, Huh7 and Huh7-SR cells were incubated with serial concentrations of Sor for 48 h. Cell growth inhibition was assessed by MTT assay. (B) Cells were incubated with Sor (10uM) and analyzed for apoptosis by FACS analysis. (C) The protein levels of p- STAT3 were determined by Western blotting (D) Cells were exposed to PH at the indicated concentrations for 48 h. DNA fragmentation was measured by cell death detection ELISA. Experiments were conducted in triplicate and mean values ± SD (bars) are shown. *p<0.05, and **p<0.01 versus control. (DOCX 111 kb)

Additional file 5: **Figure S5.** PH potentiates the antitumor effect of Sor in HepG2 and SK-Hep1 xenografts. (A) HepG2 and SK-Hep1 xenografts were treated with PH (50mg/kg) or Sor (20mg/kg) or both and tumor growth was measured every 6 days. (B) No significant change in bodyweight was observed in treated groups (C) H and E images of heart, lung, liver and spleen showing no toxic effect of with PH treatment. (D) The in vivo SHP-1 phosphatase activity in PH (50 mg/kg) or Sor (20 mg/kg) or both treated tumors. (E) The protein levels of p-STAT3 and its downstream proteins in HepG2 and SK-Hep1 xenografts in vivo were determined by western blot. (G) Immunohistochemical staining (Left panel) of CD31, pSTAT-3, cleaved caspase-3, Ki67 and TUNEL in PH treated SK-Hep1 xenografts. Magnification: ×200. Quantifications of Immunostainings (Left Panel) Quantification of CD31^+^ vessel area per total tumor area. Quantification of pSTAT-3, cleaved caspase-3, TUNEL and Ki67 cells per high power field of view. Data represents mean values from five random fields per tumor section. Scale bar, 50 μm. Experiments were conducted in triplicate and mean values ± SD (bars) are shown. *p<0.05, and **p<0.01 versus control. (DOCX 529 kb)

## Data Availability

Not applicable.

## Abbreviations

PH: Phloretin
Sor: Sorafenib
SHP-1: Src homology region 2 domain-containing phosphatase-1
STAT3: Signal transducer and activator of transcription 3
SR: Sorafenib-resistant
VEGFR2: Vascular endothelial growth factor receptor 2
MAPK: Mitogen-activated protein kinase
JAK: Janus kinase
ERK: Extracellular signal–regulated kinase;
AKT: Alpha serine/threonine-protein kinase
mTOR: mechanistic target of rapamycin
HCC: Hepatocellular carcinoma
PTP: protein tyrosine phosphatase
