# Supplementary material for: Phloretin attenuates STAT-3 activity and overcomes sorafenib resistance targeting SHP-1–mediated inhibition of STAT3 and Akt/VEGFR2 pathway in hepatocellular carcinoma
Source: Cell Commun Signal. 2019 Oct 16;17:127. doi: 10.1186/s12964-019-0430-7 (PMC6794763; doi:10.1186/s12964-019-0430-7)

Additional File5; Figure S5

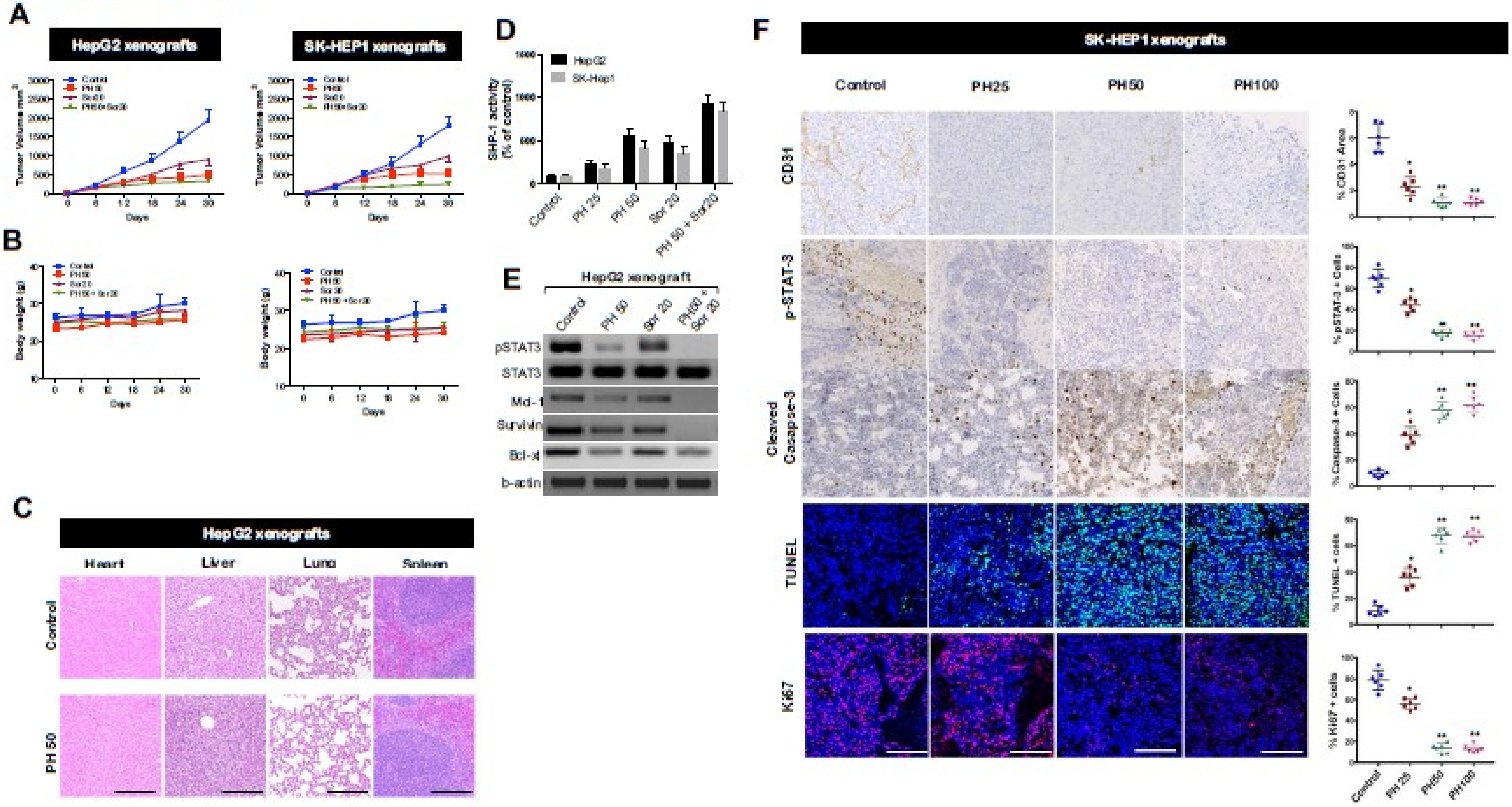

Supplement: Supplementary file 5 [file 12964_2019_430_MOESM5_ESM.docx]
